# “JUST DON’T LOOK OLD; THAT’S WHAT’LL KILL YOU”: EXAMINING OLDER WOMEN’S RESPONSE TO AGEIST BEAUTY NORMS

**DOI:** 10.1093/geroni/igae098.0014

**Published:** 2024-12-31

**Authors:** Brianna Soulie, Anne Barrett, Hope Mimbs

**Affiliations:** Florida State University, Tallahassee, Florida, United States; Florida State University, Tallahassee, Florida, United States; Florida State University, Tallahassee, Florida, United States

## Abstract

Aging beyond youth presents challenges for women, who often struggle to remain visible and valued in an ageist society. Much of the literature has focused on women’s engagement with age-related beauty work, with less attention given to the encounters with ageist beauty norms that motivate it. Studies also have given limited attention to the range of women’s responses to these norms, which can include elements of acceptance or acquiescence, as well as resistance. Our study examines these issues using in-depth interviews with 20 women, ranging in age from 57 to 83. Analyses revealed that ageist beauty norms are woven into women’s everyday interactions, often with co-workers, friends, or acquaintances. Our analyses unpack these norms to reveal the dominant age ideologies they reflect. They center on the assumption that faces and bodies that have aged beyond youth are unattractive and that the morally and practically appropriate response is to engage in beauty work to mask signs of aging. Women differed, however, in how they responded to these pressures, with some engaging in age-related beauty work and others opting out of it. Our analyses revealed that this choice often hinged on women’s perceived security in their social roles – in particular, their employment and marital or partnership statuses. While many studies have examined how women engage in beauty work to remain competitive in the youth-oriented dating and job markets, our analyses revealed how security in these realms can lead women to disengage from beauty work aimed at masking signs of aging.

